# Hexokinase 2 enhances the metastatic potential of tongue squamous cell carcinoma via the SOD2-H_2_O_2_ pathway

**DOI:** 10.18632/oncotarget.13763

**Published:** 2016-12-01

**Authors:** Wei Wang, Zhonghua Liu, Luodan Zhao, Jingjing Sun, Qianting He, Wangxiang Yan, Zhiyuan Lu, Anxun Wang

**Affiliations:** ^1^ Department of Oral and Maxillofacial Surgery, First Affiliated Hospital, Sun Yat-Sen University, Guangzhou, Guangdong, 510080, China; ^2^ Department of Stomatology, Sun Yat-Sen Memorial Hospital, Sun Yat-Sen University, Guangzhou, Guangdong, 510120, China

**Keywords:** tongue squamous cell carcinoma, hexokinase, glycolysis, metastasis, manganese superoxide dismutase (SOD2)

## Abstract

The glycolytic enzyme hexokinase (HK2), which is aberrantly expressed in various types of tumours, is associated with metastasis. However, its role in the progression and metastasis of tongue squamous cell carcinoma (TSCC) remains unclear. The results of our study showed that HK2 expression is often deregulated in TSCC patients. Increased HK2 expression was associated with tumour stage, clinical stage, lymph node metastasis, but not pathological grade, and reduced overall survival. Microarray and western blotting analyses revealed increases in HK2 expression in TSCC cells with higher metastatic potential. The following effects were observed with HK2 knockdown: inhibition of cell migration and invasion; reduced SOD2 activity and intracellular H_2_O_2_ levels; suppression of pERK1/2, Slug and Vimentin expression; and inhibition of tumour growth and lung metastasis *in vivo*. Conversely, HK2 overexpression promoted cell migration and invasion, increased SOD2 activity and intracellular H_2_O_2_, and enhanced expression of pERK1/2, Slug and Vimentin. Thus, our results demonstrate that deregulation of HK2 expression has an important function in the progression of TSCC and may serve as a biomarker of its metastatic potential in TSCC patients. HK2 enhances the metastatic potential of TSCC by stimulating the SOD2-H_2_O_2_ pathway.

## INTRODUCTION

In contrast to normal cells, most cancer cells rely on glycolysis, even under normoxic conditions; this is a metabolic phenomenon called the Warburg effect [1]. Hexokinase (HK) catalyses the essentially irreversible first step of the glycolytic pathway, whereby glucose is phosphorylated to form glucose-6-phosphate. As this product subsequently enters the glycolytic, oxidative phosphorylation (OXPHOS) or pentose phosphate pathway, HK is involved in almost all glucose metabolic processes [2]. Four isoforms of HK have been identified in mammalian tissues. HK2 is expressed mostly in embryonic tissues and in the adipose tissue and skeletal and cardiac muscles of adults [3]. Indeed, most normal mammalian tissues express very little HK2 [4]. In contrast, a high level of HK2 expression has been observed in many types of cancer, and expression is associated with poor overall survival in cancer patients [5, 6]. Patra et al. reported that HK2 plays an important role in tumour initiation and maintenance and that the HK2 gene can be systemically deleted without adverse physiological consequences. These findings suggest that HK2 deletion is an attractive potential therapeutic intervention for cancer patients [6]. The results of a recent study also suggest that HK2 expression might be closely associated with tumour metastasis [7]. To date, few studies have focused on the relationship between the level of HK2 and TSCC progression [8, 9]. Grimm et al. showed that HK2 expression was significantly increased during OSCC development [8].

TSCC, the most common type of carcinoma in the oral and maxillofacial regions, is characterized by rapid local invasion and migration. Lymph node and distant metastases are the most reliable adverse prognostic factors in patients with TSCC, and a recent study demonstrates that many factors influence the propensity of tumours to metastasize [10]. In our previous studies [11–18], we reported that abnormal expression of manganese superoxide dismutase (SOD2), microtubule-associated tumour suppressor gene (MTUS1), miR-138, miR-222 and miR-181a affect TSCC invasion and metastasis through different signalling pathways, such as the SOD2-H_2_O_2_, ERK (extracellular-regulated kinase)-Slug (Snail homolog 2, Snail2, also known as Slug) and miR138-Slug pathways. In those studies, we showed that the SOD2-dependent production of H_2_O_2_ promotes the migration and invasion of TSCC via the Snail signalling pathway [17].

To investigate the role of the glycolytic enzyme HK2 in the development of TSCC and whether HK2 promotes TSCC metastasis through the SOD2-H_2_O_2_ pathway, we here evaluate the level of HK2 expression in TSCCs using immunohistochemistry and investigate the effect of HK2 on the migratory and invasive abilities of TSCC cells *in vitro* and *in vivo*. Finally, we analyse the mechanism through which HK2 enhances TSCC metastasis. We found that deregulation of HK2 plays an important role in TSCC progression and enhances the metastatic potential of TSCC by stimulating the SOD2-H_2_O_2_ pathway.

## RESULTS

### Deregulated HK2 expression is associated with the development of TSCC and prognosis of TSCC patients

**Figure 1:**
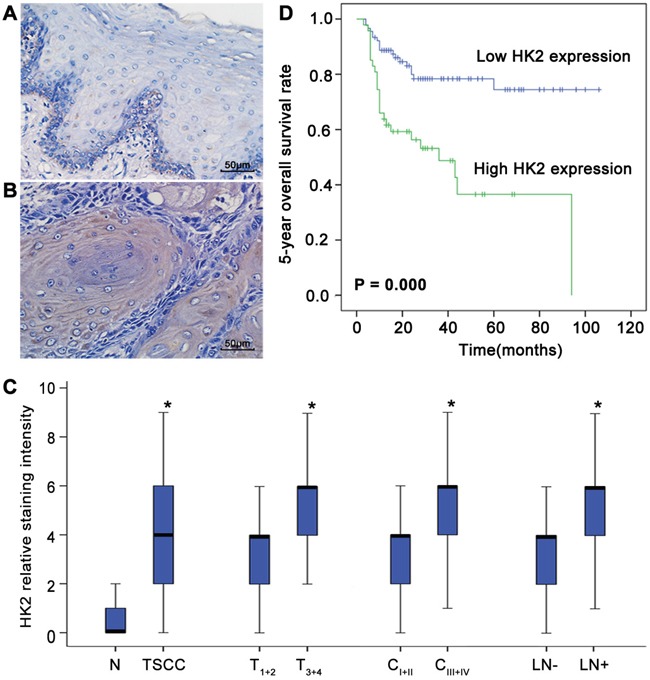
Deregulated HK2 expression is associated with the development of TSCC and prognosis of patients with TSCC HK2 expression in normal tongue tissues **A**. and TSCC tissues **B**. was analysed by IHC. Scale bar: 50 μm. **C**. Box plots demonstrate the comparative levels of HK2 expression in normal tongue tissues and TSCC tissues, in TSCC of different tumour stages and different clinical stages, and in patients with or without lymph node metastasis. The boxes represent the 25th to 75th percentiles of the observations, and the lines in the middle of the box indicate the median values. *P<0.05. **D**. Kaplan-Meier plots of the 5-year overall survival rates of patient groups, as defined by the level of TSCC HK2 expression. Differences in the survival rates of the high HK2 expression group and low HK2 expression group are significant (*P*=0.000).

To examine the relationship between deregulated HK2 expression and TSCC development, immunohistochemical analysis of HK2 expression in tissue samples obtained from 137 TSCC patients and 20 patients with normal tongue epithelium was performed. As shown in Figure 1A and 1B, HK2 was expressed in the cytoplasm; expression was rarely detectable in normal tongue tissues (Figure 1A) but was pronounced in TSCC tissues (Figure 1B). The few HK2-positive cells observed in the epithelium basal layer of normal tongue tissue suggests that HK2 might also be expressed in proliferating normal cells. Compared with normal tongue tissues, the level of HK2 expression was significantly increased in primary cancer tissues (Figure 1C). Among TSCC cases, the levels of HK2 were significantly higher in tumours of grade T_3+4_ than in those of grade T_1+2_, in tumours of grade C_III+IV_ than in those of grade C_I+II_, and in patients with lymph node metastasis than in those with no lymph node metastasis (Figure 1C and Supplementary Figure S1). No significant differences in the levels of HK2 expression with regard to age, gender or pathological grade were found (Supplementary Table S1).

To elucidate the prognostic role of the level of HK2 expression in TSCC patients, we examined the relationship between this expression level and patient outcome using the long-term follow-up period as the variable. The survival period of the high HK2 expression group was shorter than that of the low HK2 expression group (mean survival period, 46.3 vs 84.3 months). As shown in Figure 1D, a striking difference was observed for the 5-year OS of the high and low HK2 expression groups (*P*=0.000). To further evaluate the association of the HK2 expression level and clinicopathological factors with TSCC patient prognosis, univariate and multivariate analyses were conducted. As shown in Table 1, both analyses indicated that the level of HK2 expression is a prognostic factor for the 5-year OS of patients with TSCC. Thus, our findings indicate that the HK2 expression level is significantly associated with the prognosis of TSCC.

**Table 1:** Univariate and multivariate analyses of factors affecting the 5-year overall survival of patients with TSCC

Characteristic	Univariate analysis	Multivariate analysis		
	*P* value	*P* value	*HR*	*95% CI*
Age (>40 vs ≤40)	0.275	0.057	2.843	(0.970, 8.331)
Gender (Female vs Male)	0.258	0.294	1.430	(0.734, 2.788)
Pathological grade (Moderate/Poor vs Well)	0.003	0.010	2.541	(1.256, 5.143)
Tumour stage (T_1+2_ vs T_3+4_)	0.001	0.010	3.298	(1.324, 8.215)
Lymph node metastasis(Positive vs Negative)	0.022	0.180	1.654	(0.792, 3.452)
Clinical stage (C_I+II_ vs C_III+IV_)	0.007	0.421	0.679	(0.264, 1.745)
HK2 expression (Low vs High)	<0.001	0.031	2.148	(1.072, 4.307)

### HK2 expression affects the migratory/invasive capacity of TSCC cells

**Figure 2:**
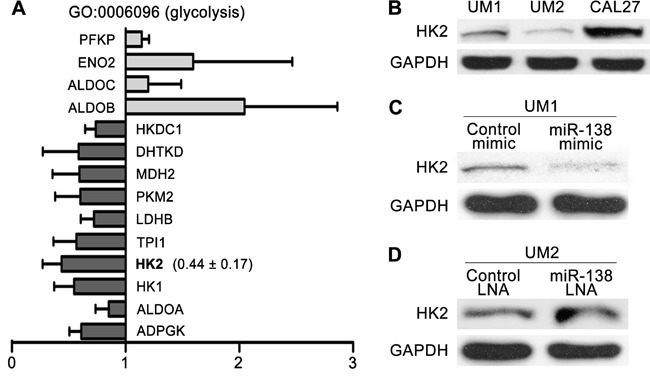
HK2 expression levels of TSCC cells with different migratory/invasive potential **A**. The expression levels of 10 genes associated with glycolysis (GO: 0006096), including HK2, were consistently reduced in TSCC cells with lower migratory/invasive potential (UM2, SCC9 and UM1 cells stably transfected with miR138 mimic). The results presented are the mean values ± standard deviation (SD). **B**. The levels of HK2 protein in UM1/UM2/CAL27 TSCC cells were evaluated by western blotting. **C-D**. Western blot showing the levels of HK2 protein in TSCC cells treated with miR-138 mimic (C) or miR-138 LNA (D).

To investigate whether the level of HK2 expression is related to the migratory/invasive potential of TSCC, microarray-based gene expression profiling was performed on three pairs of TSCC cell lines. Gene Ontology (GO) analysis revealed that expression of 10 genes associated with glycolysis (GO: 0006096) was consistently down-regulated in TSCC cells with less migratory/invasive capacity; in contrast, expression of 4 genes associated with glycolysis was consistently up-regulated. HK2 expression was consistently reduced in TSCC cells with less migratory/invasive capacity (Figure 2A). Moreover, the level of HK2 protein was increased in UM1 cells with more migratory/invasive capacity compared with that in UM2 cells with less migratory/invasive capacity (Figure 2B). When UM1 cells were transfected with the miR-138 mimic, which decreases their migration and invasion (as shown in our previous study [15]), the level of HK2 expression was notably reduced (Figure 2C). In contrast, when UM2 cells were transfected with miR-138 LNA, which increases migration and invasion [15], the level of HK2 expression was markedly increased (Figure 2D). These results reveal that the HK2 expression level is related to the migratory/invasive capacity of TSCC cells.

### HK2 enhances the migratory/invasive capacity of TSCC cells *in vitro*

**Figure 3:**
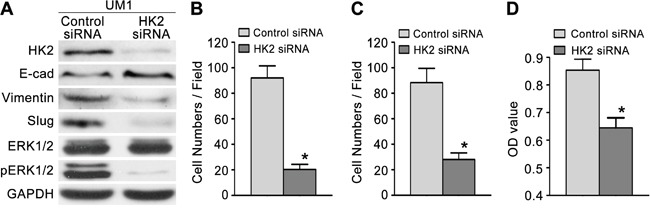
Knocking down HK2 expression inhibits TSCC cell migration and invasion **A**. Western blot displaying the expression levels of HK2, pERK1/2, ERK1/2 and EMT markers (E-cad, Vimentin, Slug) in TSCC cells transfected with HK2 siRNA. **B-C**. Knocking down HK2 expression inhibited the migration (B) and invasion (C) capacity of UM1 cells. **D**. Knocking down HK2 expression also inhibited the proliferative capacity of UM1 cells. *P<0.05.

**Figure 4:**
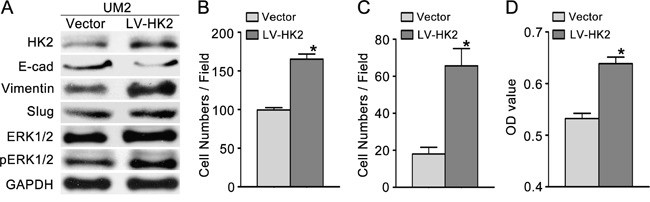
HK2 overexpression promotes TSCC cell migration and invasion **A**. Western blot showing the levels of HK2, pERK1/2, ERK1/2 and EMT markers (E-cad, vimentin, Slug) in TSCC cells infected with a lentivirus containing HK2 cDNA. **B-C**. HK2 overexpression promoted the migration (B) and invasion (C) of UM2 cells. **D**. HK2 overexpression also increased the proliferative capacity of UM2 cells. *P<0.05. LV-HK2: lentiviral constructs containing HK2 cDNA; Vector: control lentiviral construct.

To investigate the effect of HK2 on the migratory/invasive capacity of TSCC cells, we first knocked down HK2 expression using RNA interference. As shown in Figure 3A, the level of HK2 protein was significantly decreased in UM1 cells transfected with HK2 siRNA. UM1 cells transfected with HK2 siRNA displayed decreased migratory (Figure 3B and Supplementary Figure S2), invasive (Figure 3C and Supplementary Figure S2) and proliferative (Figure 3D) capacities compared with control siRNA-transfected cells. Moreover, we overexpressed HK2 in UM2 cells using a lentivirus construct containing HK2 cDNA and found that the level of HK2 protein was increased (Figure 4A). Moreover, UM2 cells transfected with the HK2 lentivirus exhibited greater migratory (Figure 4B and Supplementary Figure S2) and invasive (Figure 4C and Supplementary Figure S2) capacities compared with cells infected with the control virus. HK2 overexpression also increased the proliferative capacity of UM2 cells (Figure 4D).

### HK2 enhances TSCC migration/invasion by stimulating the SOD2-H_2_O_2_ pathway

**Figure 5:**
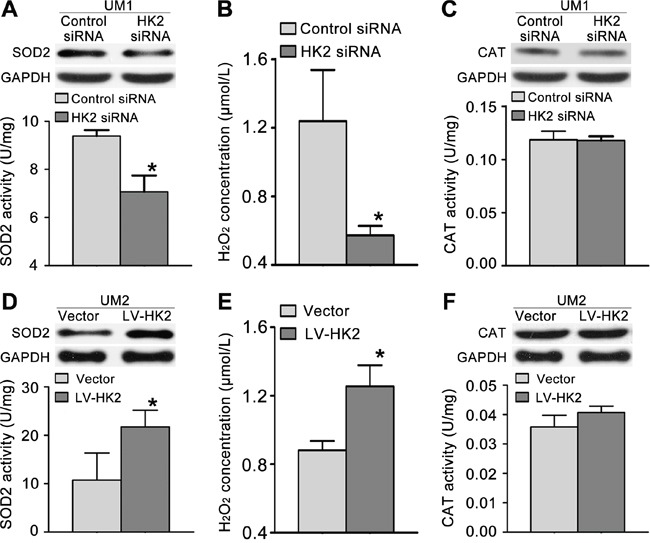
HK2 modulates TSCC cell migration/invasion through the SOD2-H_2_O_2_ pathway **A-C**. Knocking down HK2 expression inhibited SOD2 expression and activity (A) and H_2_O_2_ production (B) but did not alter catalase (CAT) expression or activity (C) in UM1 cells. **D-F**. HK2 overexpression resulted in increased SOD2 expression and activity (D) and H_2_O_2_ production (E) but did not alter catalase expression or activity (F) in UM2 cells. *P<0.05.

To investigate whether HK2 enhances the migration/invasion of TSCC cells through the SOD2-H_2_O_2_ pathway, we knocked down HK2 expression in UM1 cells using RNA interference. As shown in Figure 5A–5C, HK2 knockdown resulted in reduced SOD2 expression and activity (Figure 5A) as well as reduced H_2_O_2_ production (Figure 5B) but did not alter catalase expression or activity (Figure 5C). Knocking down HK2 expression also decreased the expression level of pERK1/2 (phosphorylated ERK1/2) and EMT (epithelial–mesenchymal transition) markers (Slug and Vimentin) and increased that of E-cadherin (E-cad) but did not notably change ERK1/2 expression (Figure 3A). In contrast, UM2 cells transfected with the HK2 lentivirus exhibited increased SOD2 expression and activity (Figure 5D) and H_2_O_2_ production (Figure 5E) but no change in catalase expression or activity (Figure 5F). HK2 overexpression in UM2 cells clearly increased expression of pERK1/2 and EMT markers (Slug and Vimentin) while decreasing that of E-cad (Figure 4A). HK2 overexpression in UM2 cells did not markedly alter expression of ERK1/2.

### Knocking down HK2 expression inhibits TSCC tumour growth and lung metastasis *in vivo*

**Figure 6:**
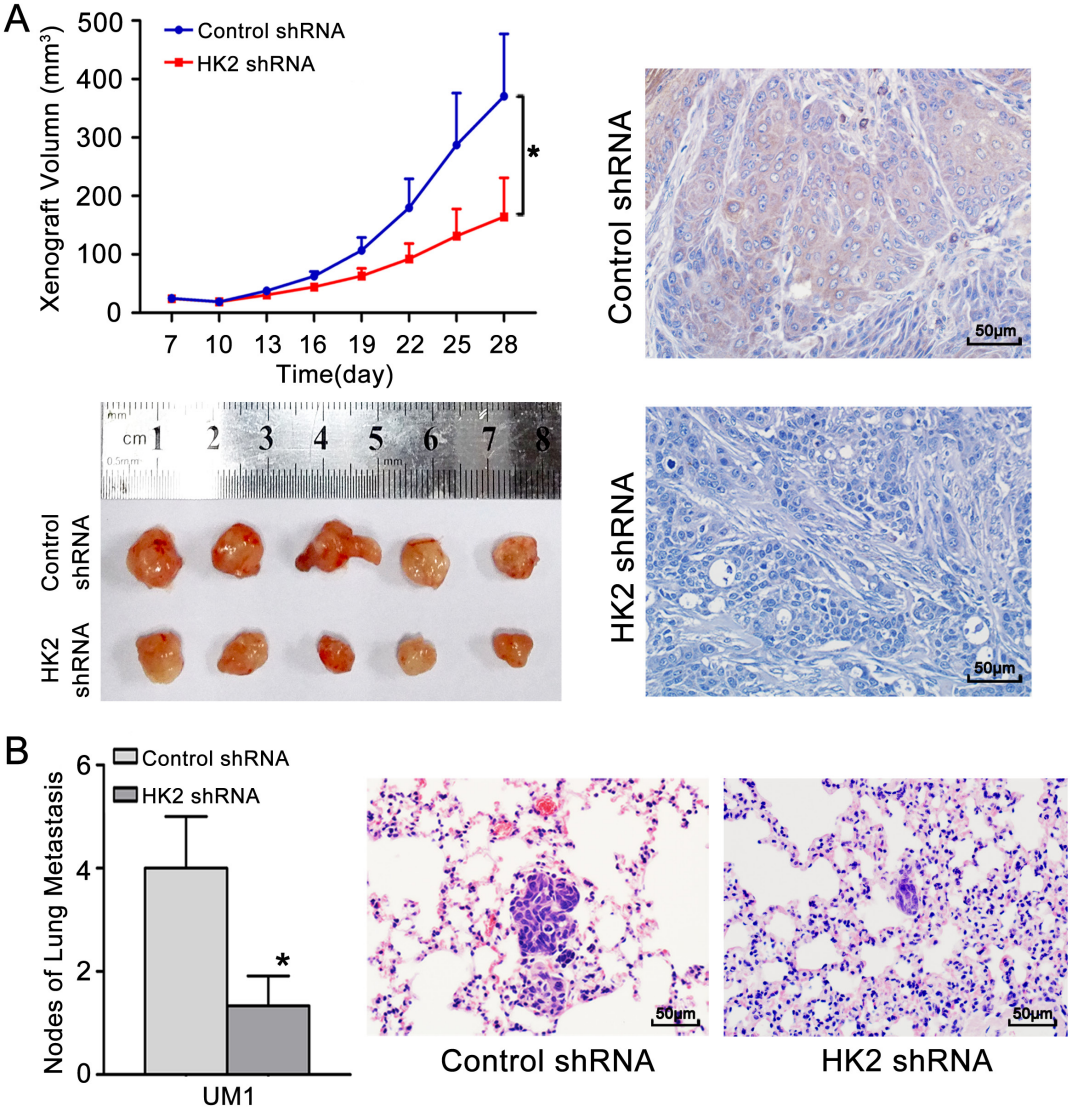
Knocking down HK2 expression inhibits TSCC xenograft growth and lung metastasis in vivo **A**. CAL27 cells stably infected with a lentivirus containing control shRNA or HK2 shRNA were inoculated subcutaneously into nude mice. Growth curves demonstrated that knocking down HK2 expression significantly inhibited CAL27 xenograft growth. IHC revealed the lower level of expression of HK2 in samples from HK2 shRNA-treated TSCC xenografts compared to control shRNA-treated TSCC xenografts. **B**. UM1 cells stably infected with a lentivirus containing control shRNA or HK2 shRNA were injected into the tail veins of nude mice. TSCC metastasis to the lungs was assessed at 6 weeks after injection. Histopathological evidence of lung metastasis (magnification 400×) and metastatic lung nodules is shown. Scale bar: 50 μm. *P<0.05.

To confirm that HK2 plays a role in the growth and metastasis of TSCC *in vivo*, the growth and lung metastasis of TSCC xenografts in nude mice were examined. CAL27 cells, which showed the highest level of HK2 expression among the tested TSCC cell lines (Figure 2B), were stably infected with a lentivirus containing HK2 shRNA and then inoculated subcutaneously into nude mice. As shown in Figure 6A, the growth of tumours derived from CAL27 cells infected with the HK2 shRNA was significantly suppressed compared with that of tumours derived from CAL27 cells infected with control shRNA. Knocking down HK2 expression inhibited tumour growth by 44.9% at day 28 after transplantation. The doubling times for tumours derived from control shRNA-infected cells and HK2 shRNA-infected CAL27 cells were 1.4 and 2.1 days, respectively. Moreover, HK2 expression (by IHC, Figure 6A) was significantly lower in samples from HK2 shRNA-treated TSCC xenografts than that in control shRNA-treated TSCC xenografts. UM1 cells stably infected with a lentivirus containing HK2 shRNA were injected into the tail veins of nude mice, and the metastatic nodules established by these cells in the lungs were confirmed histologically and counted, as in our previous study [11]. Mice injected with HK2 shRNA-infected cells exhibited a significantly reduced number of metastatic nodules compared with those injected with control shRNA-infected cells (Figure 6B).

## DISCUSSION

Among glycolytic enzymes, HK2 particularly favours aerobic glycolysis due to its intracellular localization and kinetic characteristics [19]. Many studies have reported a high level of HK2 expression in cancer cells, and this has been associated with the risk of metastasis and poor prognosis [3, 5, 7, 20]. Wolf et al. showed that HK2 is crucial for the Warburg effect to occur in human glioblastoma multiforme (GBM) [5]. In contrast to the level of HK2 in normal brains and low-grade gliomas, HK2 expression is increased in GBM tumours and is correlated with worse overall survival in patients with this disease [5].Recently, deregulated HK2 expression was also found to play a role in the pathogenesis of OSCC [8]. In the present study, we found that deregulation of HK2 expression was frequently correlated with the progression of TSCC and that increased HK2 expression was associated with lymph node metastasis and reduced 5-year OS. Up-regulated HK2 expression can thus also serve as a prognostic factor for patients with TSCC. These findings reveal that HK2 has important roles in the development and prognosis of TSCC.

Several previous studies have shown that inhibiting HK2 expression decreases the cancer cell survival rate as well as their metastatic potential [21–23]. Indeed, preventing HK2 expression with a short hairpin RNA (shRNA) impeded cancer cell survival and inhibited tumour growth in a xenograft model [5, 24]. Palmieri et al. found that *in vitro*, HK2 mRNA and protein levels were elevated in a brain metastatic derivative (231-BR) of the human breast carcinoma cell line MDA-MB-231 relative to the parental cell line (231-P); knocking down HK2 expression in 231-BR cells using a short hairpin RNA reduced their proliferation rate [7]. In this study, we found that TSCC cells with higher migratory/invasive capacity had higher levels of HK2 expression. HK2 overexpression promoted the proliferation, migration and invasion of TSCC cells, whereas knocking down HK2 expression inhibited these processes both *in vitro* and *in vivo*. These findings demonstrate that HK2 modulates the proliferative and metastatic capacities of TSCC.

Mitochondrial SOD2 efficiently converts superoxide molecules to H_2_O_2_, which can be degraded to form water and dioxygen by other antioxidant enzymes (catalase) and by non-enzymatic antioxidants. Our previous studies showed that SOD2 is deregulated during TSCC development, and SOD2-dependent production of H_2_O_2_ was found to promote the migratory and invasive capacities of TSCC and salivary adenoid cystic carcinoma (SACC) via the ERK-Snail (Slug) signalling pathway [16, 17, 25–28]. Moreover, we also found that Bmi1-mediated migration and invasion of TSCC involves cancer stem-like cells and the SOD2-H_2_O_2_ pathway [29]. In the present study, we found that knocking down HK2 expression resulted in decreased levels of SOD2 activity, intracellular H_2_O_2_ and pERK1/2 and Slug expression. In contrast, HK2 overexpression resulted in the opposite effects. These results implicate HK2 in the modulation of the migratory/invasiveness potentials of TSCC through the SOD2-H_2_O_2_ pathway.

According to our results, deregulating the expression of the glycolytic enzyme HK2 has an important role in the development of TSCC and is associated with poor prognosis in patients with TSCC. Deregulated HK2 expression contributed to increasing the metastatic potential of TSCC cells *in vitro* and *in vivo*, and HK2 enhanced the migration/invasion of these cells by stimulating the SOD2-H_2_O_2_ pathway. These findings suggest that inhibiting HK2 expression is an attractive potential therapeutic intervention for patients with TSCC.

## MATERIALS AND METHODS

### Patient samples

For this retrospective study, we collected tissue samples from 137 patients with TSCC who had undergone radical resection without preoperative chemotherapy or radiotherapy and 20 matching adjacent non-cancerous samples (normal tongue tissues) from patients undergoing surgery at First Affiliated Hospital of Sun Yat-Sen University (June 2004-September 2014). The clinical characteristics of the patients are summarized in Supplementary Table S2. Survival was calculated from the date of diagnosis to the date of the latest follow-up (or death) (2014-09-01). This study was approved by the ethics committee of Sun Yat-Sen University (2016074).

### Immunohistochemical staining

Immunohistochemistry (IHC) was used to detect HK2, as previously described [12]. Briefly, paraffin sections were deparaffinized using xylene and rehydrated using a series of alcohol solutions. Antigen retrieval was performed by treating the sections with boiling citrate buffer (pH 6.0) for 4 min in a pressure cooker. Endogenous peroxidase activity was blocked by treatment with 3% H_2_O_2_ for 15 min. The sections were then incubated with an anti-HK2 antibody (Atlas, 1:250) overnight at 4°C. The sections were incubated with the secondary antibody using a MaxVision™ HRP-Polymer anti-Rabbit IHC Kit (Maixin, Fuzhou, China); colour was developed using a DAB Horseradish Peroxidase Colour Development Kit (Maixin, Fuzhou, China), and the sections were counterstained with haematoxylin. The degree of immunostaining was scored according to both the proportion of positively stained tumour cells and the staining intensity, as described previously [30]. We evaluated HK2 expression by determining the staining index (staining intensity score × proportion of positive tumour cells), which resulted in scores of 0, 1, 2, 3, 4, 6 and 9. An optimal cut-off value (median) was identified, with a staining index score of >4 defining a high level of HK2 expression and a staining index score of ≤4 defining a low level of HK2 expression in the tumours.

### Cell culture and transfection

Human TSCC cell lines (UM1, UM2, CAL27) were maintained in DMEM supplemented with 10% foetal bovine serum, 1000 U/ml penicillin and 500 mg/ml streptomycin in a 37°C incubator containing 5% CO_2_. UM1 and UM2 are paired cell lines from a single patient with TSCC that exhibit different metastatic potentials [31]. To knock down HK2 expression, cells were transfected with HK2 siRNA or control siRNA (Ribobio, Guangzhou, China) using the Lipofectamine™ RNAiMAX transfection reagent (Invitrogen, CA, USA) according to the manufacturer's instructions. Three sequences of HK2 siRNA were tested, and the sequence with the strongest knockdown effect was chosen for further use. For HK2 overexpression, a lentivirus construct containing HK2 cDNA (NM_000189.4) (Chemgene, Shanghai, China) was packaged into 293T cells using ViraPower Mix (Invitrogen, CA, USA). Lentivirus infection of cells (MOI=1:50) was performed in the presence of 5 μg/ml polybrene (Sigma-Aldrich, MO, USA). miR-138 mimic, control mimic, a locked nucleic acid (LNA) inhibitor of miR-138 (anti-miR-138 LNA) and a control LNA (Genepharma, Shanghai, China) were also transfected into appropriate cells, and the cells were harvested for functional analysis after 48 h. The sequences of the HK2 siRNA, miR-138 mimic and miR-138 LNA are shown in Supplementary Table S3.

### *In vitro* cell migration/invasion assays

Transwell assays were performed to assess cell migratory and invasion capacities using BD BioCoat Control Cell Culture Inserts and BD BioCoat BD Matrigel^TM^ Invasion Chambers, respectively [26]. Briefly, cells were seeded in the upper Boyden chambers of the cell culture inserts. After 24 h, the cells that had adhered to the lower membrane were stained with DAPI in the dark and then visualized and counted. Images of three random fields were captured at 200× magnification under a microscope. The number of cells on the bottom surface in each group was compared.

### Cell proliferation assays

Cell proliferation was evaluated after 24 h using a modified Cell Counting Kit-8 (CCK8) assay (Fanbo, Beijing, China) according to the manufacturer's instructions [30]. The absorbance (optical density, OD) value at 450 nm was determined using a plate reader.

### Western blotting analysis

Western blotting analysis was performed as described previously [17] using antibodies specific for E-cad, Vimentin, Slug, ERK1/2, pERK1/2, SOD2, GAPDH (Cell Signaling Technology, MA, USA), HK2 and catalase (Sigma-Aldrich, MO, USA). GAPDH was used as the control (Cell Signaling Technology).

### SOD2/catalase activity and intracellular H_2_O_2_ concentration

The level of SOD2 activity was determined using a WST-8-containing Cu/Zn-SOD and Mn-SOD Assay Kit (Beyotime, China) according to the manufacturer's instructions [17]. Catalase activity was evaluated using a catalase assay kit (Molecular Probes, USA) according to the manufacturer's instructions [17]. The intracellular H_2_O_2_ concentration was determined using a PeroXOquant quantitative peroxide assay kit (Pierce, IL, USA) according to the manufacturer's instructions [17].

### Gene expression profile by microarray analysis

mRNA expression profiles of three pairs of TSCC cell lines with different metastatic potential (SCC9 cells transfected with miR-138 mimic vs those transfected with control mimic, UM1 cells transfected with miR-138 mimic vs those transfected with control mimic and UM2 cells vs UM1 cells) were determined. Human Genome U133A Array (HG U133A, Affymetrix, Santa Clara) was used for gene expression analysis as previously reported [32].

### Tumorigenesis and metastasis assay in nude mice

To investigate the effect of HK2 on tumorigenesis and metastasis *in vivo*, UM1 or CAL27 cells stably infected with a lentivirus containing HK2 shRNA (Genechem, Shanghai, China) were collected and re-suspended in DMEM/F12 for tumorigenesis and metastasis assays, which were performed as described in our previous report [11]. Treated CAL27 cells (5×10^6^/0.2 ml) were inoculated subcutaneously into both flanks of 4-wk-old male BALB/c nude mice (purchased from the Institute of Zoology, Chinese Academy of Sciences, Guangzhou), and the resulting xenografts were measured using a calliper beginning at 1 week after inoculation. The tumour volumes were calculated as ½length × width^2^; a tumour growth curve (y=Ae^kday^) and the tumour doubling time (ln2/k) were obtained as previously described [11]. For metastasis assays, treated UM1 cells (1×10^6^/0.2 ml) were injected into the tail veins of BALB/c nude mice. The animals were sacrificed 8 weeks later, and metastatic tumours in the lungs were assessed as previously described [11]. None of the mice showed any notable toxic effect or loss of body weight during the experimental period. This animal study was approved by the ethics committee of our institution (2016047).

### Statistical analysis

All of the statistical analyses were performed using Statistical Package for the Social Sciences (SPSS, Chicago, IL), version 19.0. The χ^2^ test was used to analyse the relationship between the level of gene expression and the clinicopathological characteristics of TSCC patients. Survival curves were plotted using the Kaplan-Meier method and compared using the log-rank test. Cox regression was used for univariate and multivariate analyses. Student's t test was used to analyse the significance of differences between two groups. In all cases, significance was defined as *P* values less than 0.05. All *P* values were two-sided.

## SUPPLEMENTARY MATERIALS FIGURES AND TABLES


