# Supplementary material for: Hexokinase 2 enhances the metastatic potential of tongue squamous cell carcinoma via the SOD2-H2O2 pathway
Source: Oncotarget. 2016 Dec 1;8(2):3344–54. doi: 10.18632/oncotarget.13763 (PMC5356886; doi:10.18632/oncotarget.13763)
Supplement: Supplementary file 1 [file oncotarget-08-3344-s001.pdf]

Hexokinase 2 enhances the metastatic potential of tongue squamous cell carcinoma via the SOD2-H_2O_2 pathway

SUPPLEMENTARY FIGURES AND TABLES

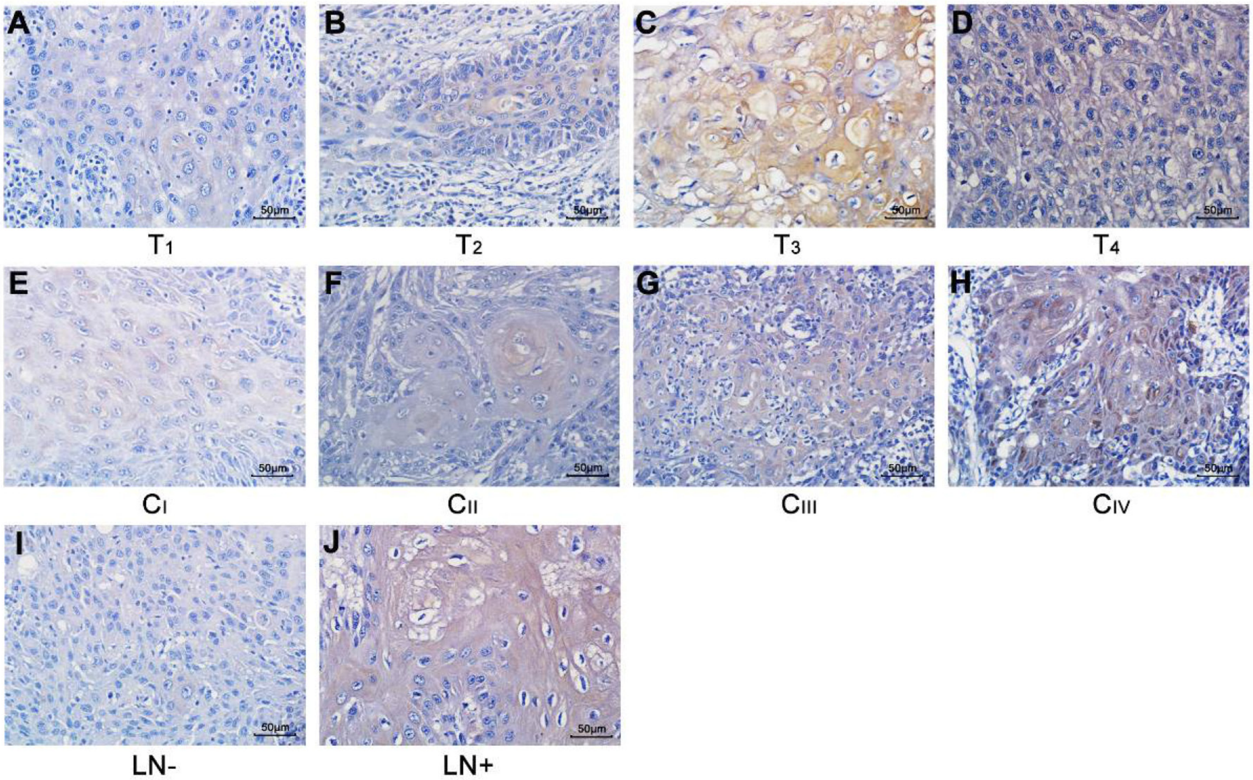

Supplementary Figure S1: HK2 expression in TSCC patients with different tumour stage, clinical stage and lymph node metastasis. Immunohistochemical staining shows representative HK2 expression in T_1, T_2, T_3, T_4, C_I, C_{II}, C_{III}, and C_{IV} and in a patient with LN- and a patient with LN+. Scale bar: 50 µm.

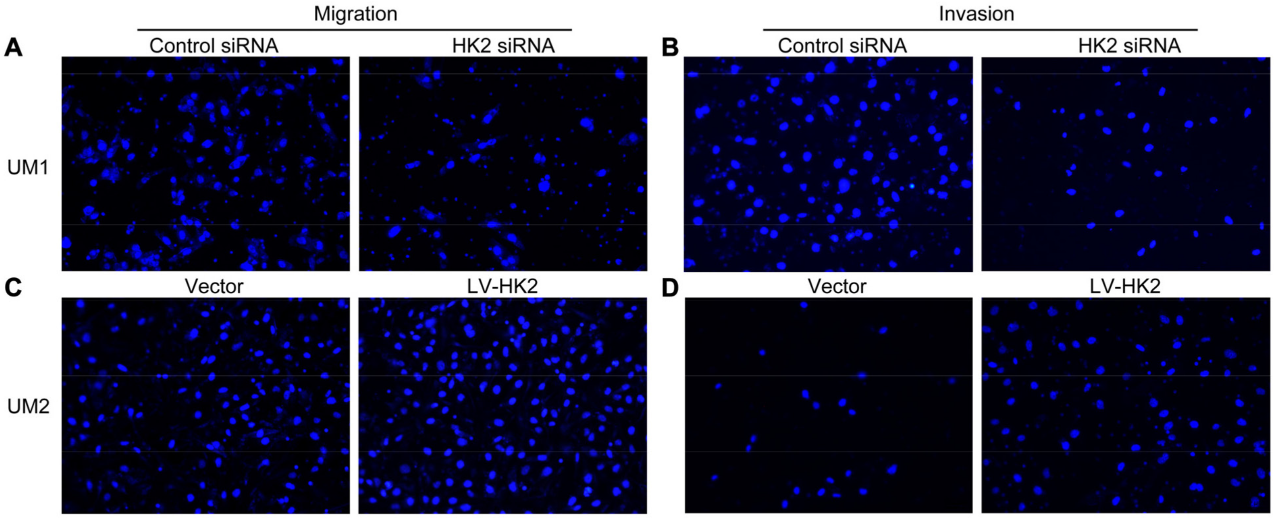

Supplementary Figure S2: Migration and invasion capacities of UM1 and UM2 cells after knockdown or overexpression of HK2. Migration and invasion capacities of UM1 and UM2 cells were assessed using a transwell migration and invasion assay. HK2 knockdown inhibited the migration **A.** and invasion **B.** of UM1 cells. HK2 overexpression significantly increased the migration **C.** and invasion **D.** of UM2 cells. LV-HK2: lentiviral constructs containing HK2 cDNA; Vector: control lentiviral construct.

Supplementary Table S1: The relationship between HK2 expression and the clinicopathological characteristics of patients with TSCC

Characteristics		HK2		*P* value
		Low	High	
Gender	Male	62	27	0.183
	Female	28	20	
Age (years)	≤40	10	8	0.331
	>40	80	39	
Tumour stage	T_{1+2}	75	24	<0.001
	T_{3+4}	15	23	
LN metastasis	Negative	72	21	<0.001
	Positive	18	26	
Clinical stage	C_{I+II}	64	16	<0.001
	C_{III+IV}	26	31	
Pathological grade	Well	44	15	0.057
	Moderate/Poor	46	32	

LN: Lymph node.

Supplementary Table S2: Clinicopathological characteristics of patients with TSCC

		TSCC	Normal
Gender	Male: n (%)	89(64.96)	11(55.00)
	Female: n (%)	48(35.04)	9(45.00)
Age	Median (range)	57(27-84)	52(37-78)
Tumour stage	T_{1+2}: n (%)	100(72.99)	
	T_{3+4}: n (%)	37(27.01)	
LN metastasis	Negative: n (%)	93(67.88)	
	Positive: n (%)	44(32.12)	
Clinical stage	C_{I+II}: n (%)	80(58.39)	
	C_{III+IV}: n (%)	57(41.61)	
Pathological grade	Well: n (%)	59(43.07)	
	Moderate/Poor: n (%)	78(56.93)	

Supplementary Table S3: Sequences of HK2 siRNA and miR-138

	Sequence
HK2 siRNA	5'- GUGGACAGGAUACGAGAAAdTdT-3'
Control siRNA	5'-UUCUCCGAACGUGUCACGUTT-3'
Control mimic	5'-UUCUCCGAACGUGUCACGUTT-3'
miR-138 mimic	5'-AGCUGGUGUUGUGAAUCAGGCCG-3'
Control LNA	5'-CAGUACUUUUGUGUAGUACAA-3'
miR-138 LNA	5'-CGGCCUGAUUCACAACACCAGCU-3'
